# Quantifying blood-spinal cord barrier permeability after peripheral nerve injury in the living mouse

**DOI:** 10.1186/1744-8069-10-60

**Published:** 2014-09-13

**Authors:** Lindsay S Cahill, Christine L Laliberté, Xue Jun Liu, Jonathan Bishop, Brian J Nieman, Jeffrey S Mogil, Robert E Sorge, Catherine D Jones, Michael W Salter, R Mark Henkelman

**Affiliations:** Mouse Imaging Centre, Hospital for Sick Children, 25 Orde Street, Toronto, Ontario Canada; Program in Neurosciences and Mental Health, The Hospital for Sick Children, Toronto, Ontario Canada; Department of Psychology, McGill University, Montreal, Quebec Canada; Alan Edwards Centre for Research on Pain, McGill University, Montreal, Quebec Canada; Department of Psychology, University of Alabama, Birmingham, Alabama USA

**Keywords:** Blood-spinal cord barrier permeability, Dynamic contrast-enhanced MRI, Gd-DTPA, Mouse, Neuropathic pain, Peripheral nerve injury, Spinal cord

## Abstract

**Background:**

Genetic polymorphisms, gender and age all influence the risk of developing chronic neuropathic pain following peripheral nerve injury (PNI). It is known that there are significant inter-strain differences in pain hypersensitivity in strains of mice after PNI. In response to PNI, one of the earliest events is thought to be the disruption of the blood-spinal cord barrier (BSCB). The study of BSCB integrity after PNI may lead to a better understanding of the mechanisms that contribute to chronic pain.

**Results:**

Here we used in vivo dynamic contrast-enhanced magnetic resonance imaging (DCE-MRI) to establish a timecourse for BSCB permeability following PNI, produced by performing a spared nerve injury (SNI). From this longitudinal study, we found that the SNI group had a significant increase in BSCB permeability over time throughout the entire spinal cord. The BSCB opening had a delayed onset and the increase in permeability was transient, returning to control levels just over one day after the surgery. We also examined inter-strain differences in BSCB permeability using five mouse strains (B10, C57BL/6J, CD-1, A/J and BALB/c) that spanned the range of pain hypersensitivity. We found a significant increase in BSCB permeability in the SNI group that was dependent on strain but that did not correlate with the reported strain differences in PNI-induced tactile hypersensitivity. These results were consistent with a previous experiment using Evans Blue dye to independently assess the status of the BSCB permeability.

**Conclusions:**

DCE-MRI provides a sensitive and non-invasive method to follow BSCB permeability in the same group of mice over time. Examining differences between mouse strains, we demonstrated that there is an important genetically-based control of the PNI-induced increase in BSCB permeability and that the critical genetic determinants of BSCB opening after PNI are distinct from those that determine genetic variability in PNI-induced pain hypersensitivity.

**Electronic supplementary material:**

The online version of this article (doi:10.1186/1744-8069-10-60) contains supplementary material, which is available to authorized users.

## Background

Peripheral nerve injury (PNI) is estimated to affect 3-8% of people worldwide and often leads to chronic neuropathic pain [1]. The neuropathic pain phenotype varies widely in both humans and rodent strains, with genetic polymorphisms, gender and age all influencing the risk of developing and the intensity of persistent pain [2, 3]. In response to PNI, a cascade of cellular and molecular changes occurs, with disruption of the blood-spinal cord barrier (BSCB) thought to be one of the earliest events. The BSCB is a network of endothelial cells joined by tight junctions that acts as an interface protecting the spinal cord parenchyma from the circulation and the periphery. Dysfunction of the BSCB may allow penetration of inflammatory cells (e.g. monocytes) and immune cells (e.g. T cells) and is thought to play an important role in disorders such as traumatic brain/spinal cord injury, stroke and neurodegeneration [2, 4]. The study of BSCB integrity after PNI may lead to the development of a better understanding of the mechanisms that contribute to chronic pain.

Using intravenously administered Evans Blue dye, we have shown PNI injury causes delayed opening of the BSCB, triggered by discharge activity in TRPV1-expressing C-fibers [5]. The use of tracers such as Evans Blue to assess BSCB integrity requires a cross-sectional study design. Alternatively, in vivo dynamic contrast-enhanced magnetic resonance imaging (DCE-MRI) provides a non-invasive and quantitative technique for assessing BSCB permeability. The BSCB is normally impermeable to paramagnetic MR contrast agents such as Gd-DTPA. If the BSCB is disrupted, Gd-DTPA can leak into the spinal cord, causing it to appear hyperintense in T1-weighted MR images. This technique has been used to investigate BSCB disruption following mechanical spinal cord injury in the rat [6–10] and mouse [11], in animal models of amyotrophic lateral sclerosis [12] and in mice with experimental autoimmune encephalomyelitis (EAE) [13, 14]. An increased BSCB permeability has been correlated to behavioural metrics such as deteriorated motor function [7, 9].

In the present study, we performed spared nerve injury (SNI) in mice, a robust model in which the tibial and common peroneal nerves are injured and that closely mimics many clinical features of neuropathic pain [15, 16]. Using in vivo DCE-MRI, we examined BSCB integrity in a group of SNI and control mice over time. After establishing a timecourse for BSCB permeability, we investigated whether BSCB opening is necessary for pain hypersensitivity. A common symptom of neuropathic pain is hypersensitivity to normally innocuous stimuli, a phenomenon known as mechanical allodynia [17]. A recent study found significant inter-strain differences in tactile hypersensitivity in 18 inbred strains of mice after PNI [18]. Using a genome-wide linkage analysis, they identified a haplotype in the *P2RX7* gene that affects chronic pain sensitivity in both mice and humans. Based on this work, we chose four inbred, and one outbred (CD-1), mouse strains that spanned the range of pain hypersensitivity, from least (B10) to most (BALB/c) allodynia, and used DCE-MRI to look at inter-strain differences in BSCB permeability.

## Results

**Figure 1:**
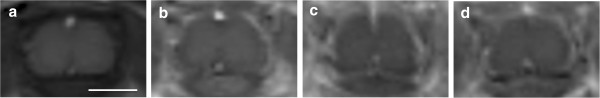
**Transverse T1-weighted MR images of the mouse spinal cord. (a)** A BALB/c mouse from the control treatment group before injection of Gd-DTPA and **(b)** after injection of Gd-DTPA; **(c)** a control CD-1 mouse and **(d)** a SNI CD-1 mouse at 29.5 hours after the surgery. These images illustrate the inherently low spatial resolution of in vivo MRI measurements in small animals such as mice. Scale bar = 1 mm.

We assessed the integrity of the BSCB by administering the MR contrast agent Gd-DTPA (Magnevist). A compromised BSCB permits the leakage of contrast agent into the spinal cord and the increase in signal intensity in T1-weighted MR images is indicative of the concentration of contrast agent in the tissue, providing a relative measure of BSCB permeability [19]. The periphery of the entire spinal cord, as well as the boundaries of the grey and white matter within the anterior of the lumbar cord, are readily delineated on a conventional in vivo T1-weighted MRI scan with 100 μm resolution (Figure 1). This quality of anatomical definition resulted in a low interobserver variability of less than 2% for the measurement of normalized spine signal intensity across all studies. Representative transverse slices through the lumbar spinal cord before and after administration of the contrast agent are shown in Figure 1a and 1b. Before administration of the contrast agent, there was no difference in the normalized spine signal intensity between treatment groups (SNI or control), over time for the longitudinal study and between mouse strains for the study of strain differences (Additional file 1). Following successful injection of the contrast agent, the surrounding cerebrospinal fluid (CSF) and tissue consistently showed elevated signal intensity. In addition, a weak positive correlation was found between the normalized spine signal intensity and normalized muscle signal intensity (adjusted R^2^ = 0.27). Thus, a calibration exercise was performed to account for factors that could contribute to the variability in the normalized spine signal intensity (i.e. differences in the tip angle of the magnetization). Here, correcting for the correlation between spinal cord signal and muscle did not reduce the variability in the data so it was not included as a factor. In all of the studies, signal enhancement following Gd-DTPA injection was found throughout the spinal cord but was more pronounced at the periphery of the cord.

### Longitudinal Study: BSCB permeability is increased in CD-1 mice following SNI

**Figure 2:**
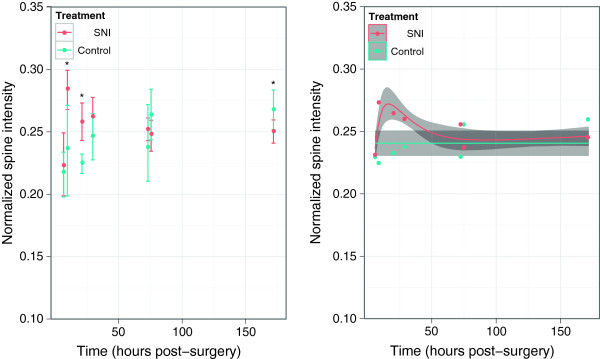
**SNI causes delayed opening of the BSCB in CD-1 mice.** A longitudinal study using DCE-MRI to follow BSCB permeability over time (hours post-surgery) in SNI (red) and control (blue) treatment groups. Left panel shows the mean data averaged over the thoracic and lumbar ROIs. The error bars represent 90% confidence intervals determined by bootstrap resampling of ROI intensity values at each time point; *p < 0.05 compared to controls. Right panel shows the data fitted with a linear mixed effects model, assuming a constant intensity for control mice and modeling intensity deviations in SNI mice with a natural spline consisting of two internal knot points (after logarithmically scaling time). The mean data points are shown after removal of per subject random effects and the grey shaded regions illustrate 90% confidence intervals determined by bootstrap resampling of subjects.

Representative transverse slices through the lumbar spinal cord in control and SNI CD-1 mice are shown in Figure 1c and 1d. Longitudinal assessment of the permeability of the BSCB in CD-1 mice revealed a significant difference in normalized spine signal intensity over time between the SNI and control groups (p = 3.8×10^−6^, Kruskal-Wallis test, Figure 2 right). In the SNI group, there was an increase in normalized spine signal intensity at 9.5 and 21 hours after surgery as compared with controls (p = 0.0058 and 0.00026 respectively, *t*-test) (Figure 2 left). The increase in signal intensity was found in both the thoracic and lumbar segments of the spinal cord even though the injury was localized to the primary afferent neurons in the lumbar segment. However, by 29.5 hours after surgery the signal intensity in the SNI group no longer differed from that of controls and there was a small but significant decrease by 172 hours (p = 0.031, *t*-test). From this longitudinal study, we conclude that the BSCB permeability has a delayed onset and that the increase in permeability is transient, returning to control levels 29.5 hours after the surgery.

### Strain Differences: BSCB permeability is increased in both CD-1 and A/J mice following SNI

**Figure 3:**
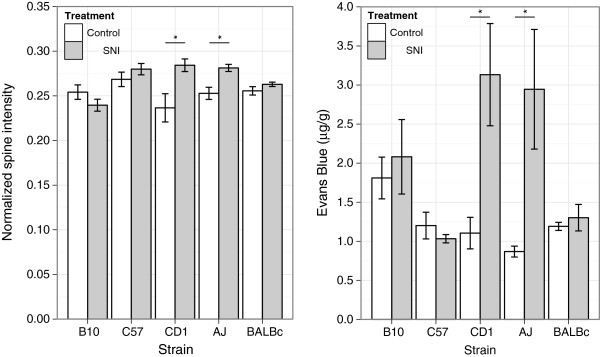
**Strain-related differences in BSCB permeability.** Left panel shows normalized spine signal intensity in SNI (grey bars) and control (white bars) treatment groups at approximately 12 hours after surgery (n = 4–6 per group, except for CD-1: n = 2–3 per group). Right panel shows accumulation of Evans Blue dye in a separate cohort of SNI (grey bars) and control (white bars) treatment groups 24 hours after surgery (n = 5 per group). The mouse strains are ordered from least to most mechanical hypersensitivity after PNI. Data are presented as mean ± SEM; *p < 0.05 compared to controls.

**Figure 4:**
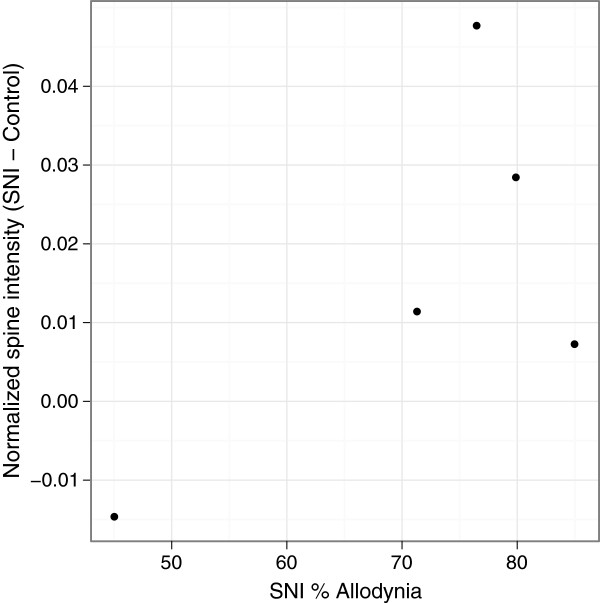
**Strain differences in BSCB permeability did not correlate with strain differences in PNI-induced tactile hypersensitivity.** Plot of the normalized spine signal intensity difference between SNI and control with the percentage of the maximum possible allodynia in five mouse strains (B10, C57BL/6J, CD-1, A/J and BALB/c) (n = 6 per group, except CD-1: n = 11 per group) (p = 0.232).

Based on the peak in normalized spine signal intensity in the longitudinal study using CD-1 mice, we chose a time point of approximately 12 hours after the surgery to investigate whether there is an effect of strain on the BSCB permeability. Figure 3 left shows the normalized spine signal intensity in five strains (B10, C57BL/6J, CD1, A/J and BALB/c), after administration of Gd-DTPA. The mouse strains are ordered from least to most PNI-induced allodynia, determined by their hindpaw withdrawal thresholds to von Frey filament stimulation (Figure 4). Overall, there was a significant increase in normalized spine signal intensity in the SNI group (treatment: p = 3.1×10^−4^, two-way ANOVA) that was dependent on strain (strain: p = 0.010, two-way ANOVA) and had a significant interaction between treatment group and strain (treatment x strain: p = 0.0016, two-way ANOVA). In addition to the increase in signal intensity in the CD-1 cohort observed in the longitudinal study (p = 0.0058, *t*-test), the A/J mice also showed a significant increase in BSCB permeability compared to controls (p = 0.0026, *t*-test). This result was consistent with a separate experiment using the Evans Blue assay to independently assess the status of the relative BSCB permeability in the same five strains of mice (Figure 3 right). There was a statistically significant increase in Evans Blue dye in the SNI group (treatment: p = 0.001, two-way ANOVA) that was dependent on strain (strain: p = 0.028, two-way ANOVA) and had a significant interaction between treatment group and strain (treatment x strain: p = 0.005, two-way ANOVA). The SNI group for the CD-1 and A/J mice showed the largest effects and a significant increase in accumulation of the Evans Blue dye as compared with controls (p = 0.018 and 0.027 respectively, *t*-test).

## Discussion

The BSCB is highly restrictive and both Gd-DTPA and Evans Blue bound to albumin are of sufficient molecular size to be impermeable to the intact BSCB (0.94 and 68 kDa respectively). If the BSCB is disrupted, these agents diffuse into the spinal cord parenchyma with no specific tissue affinity. Administration of Gd-DTPA is a non-invasive and quantitative method for measuring BSCB permeability while the use of Evans Blue is primarily a qualitative histological technique. There are several other molecules, outside of the size range of Gd-DTPA and Evans Blue, which have been used to study the permeability of the BSCB. These include small molecular tracers such as [^14^C]-alpha-aminoisobutyric acid (AIB, 0.1 kDa) [20] and large proteins such as immunoglobulin G (IgG, 160 kDa) [13].

Compared to the use of traditional invasive or terminal histological techniques, longitudinal DCE-MRI studies provide a probe of the temporal and spatial changes in the BSCB permeability within a single animal. There are several longitudinal studies in the literature that have used DCE-MRI to examine BSCB permeability in rodents. Our observation of increased BSCB disruption in the white matter compared to the grey matter is consistent with the work of Schellenberg et al. in a model of EAE [13]. This may reflect the fact that damage to the white matter following spinal cord injury is thought to be responsible for persistent functional loss [21]. Interestingly, the magnitude of the increases in BSCB permeability that we observed in this study (<20%) was quite subtle in comparison to other reports. For example, in mouse models of EAE, changes of greater than 60% have been observed [13, 14]. This is consistent with EAE as a direct insult to the spinal cord while changes in the cord following PNI is secondary to pain.

The finding of a delay in the opening of the BSCB and the return to control levels in CD-1 mice is consistent with our previous work in rats following SNI [5]. This cross-sectional design revealed a peak in Evans Blue dye at 24 hours after surgery. The fact that we observed a peak in signal intensity at an earlier time point could be attributed to the fact that Gd-DTPA has a significantly lower molecular weight than Evans Blue bound to albumin (0.94 and 68 kDa respectively). Thus, MR contrast agents are more sensitive to detecting early BSCB breakdown than histological techniques such as the Evans Blue assay. This difference could also be explained by the species differences since BSCB permeability has been reported to be greater in rats than in mice [22]. This was consistent with our examination of strain differences, where the largest accumulation of Evans Blue dye was 3.1 μg/g in the CD-1 mice compared to approximately 10 μg/g in rats [5].

Also consistent with our previous findings [5], we observed here that the increase in BSCB permeability measured by Gd-DTPA was along the spinal cord and was not limited to the lumbar segments. Thus in both rats and mice PNI causes an increase in BSCB permeability that extends well beyond the central termination of the injured primary afferent neurons. Previously, we had found that in rats the increase in permeability extends into regions of the brain as well as the spinal cord [5]. Given our previous findings, we expect that the blood–brain barrier permeability as well as the BSCB permeability were increased by PNI in mice. The widespread increase in CNS vascular permeability might involve production and release of a humoral mediator, or mediators, that act on the cellular and/or intercellular elements that maintain the intact barrier [5]. Alternatively, there might be spread of BSCB permeability from an initiation site in the lumbar spinal cord or central release of diffusible mediator(s) that circulate within the cerebrospinal fluid. The widespread increase in permeability contrasts with the activation of microglia cells following PNI [23], cells that are key intermediaries in PNI-induced pain hypersensitivity [24].

We found concordance between the MRI and Evans Blue results in terms of which strains showed PNI-induced significant increase in BSCB permeability. However, the strain differences did not correlate with strain differences in PNI-induced tactile hypersensitivity (Figure 4) [18]. For example, BALB/c mice showed the highest tactile hypersensitivity in this group yet there was no significant difference in normalized spine signal intensity or accumulation of Evans Blue in the SNI treatment group compared to controls. Thus, an increase in BSCB permeability after PNI might not be required for PNI-induced tactile hypersensitivity. Although, it is possible that there was PNI-induced opening of the BSCB in the other strains but that was not detected by the single-time point measurement we used. Evaluating this possibility would require a complete longitudinal study for each strain.

Nevertheless, the differences we did observe between the mouse strains, even though only a small number of strains were examined, demonstrate that there is important genetically-based control of the PNI-induced increase in BSCB permeability. Moreover, our findings suggest that the critical genetic determinants of BSCB opening after PNI are distinct from those that determine genetic variability in PNI-induced pain hypersensitivity. Identifying the critical genes controlling variability in BSCB opening after PNI will require larger numbers of inbred strains than used currently and quantitative trait locus mapping, as has been done for PNI-induced pain hypersensitivity (e.g. [18]) and other complex traits. Finally, the mice included in this study were all male and it would be valuable to investigate whether our strain-related findings are consistent in female mice and how BSCB permeability differs with gender since it is well-known that gender is a key factor for chronic neuropathic pain.

## Conclusions

Here, we demonstrate that DCE-MRI is a safe, non-invasive and quantitative method to follow BSCB permeability in the same group of mice over time. After PNI, BSCB opening is delayed by several hours, and is transient lasting just over one day before returning to control levels. We found strain-related differences in PNI-induced increase BSCB permeability. The between-strain changes in BSCB permeability did not correlate with behavioural measures of pain hypersensitivity, suggesting that there are critical genetic determinants of BSCB opening after PNI which are distinct from those that determine genetic variability in PNI-induced pain hypersensitivity. Thus, DCE-MRI provides a sensitive and non-invasive measure of BSCB permeability that will be useful for tracking cord changes in chronic neuropathic pain.

## Methods

### Animals

Two cohorts of male Crl:CD1(ICR) (CD-1) mice (six SNI and four controls at 4–5 weeks of age) from Charles River Laboratories (St. Constant, QC, Canada) were imaged longitudinally. MRI was performed at 6.5, 9.5, 21, 29.5, 73, 75.5 and 172 hours after surgery. To explore strain related effects, B10.D2-Hc^o^H2^d^H2-T18^c^/oSnJ (B10), C57BL/6J, A/J and BALB/c mice from Jackson Laboratories (Bar Harbor, ME, USA) were imaged at one time point, approximately 12 hours after surgery (six SNI and four controls at 5–12 weeks of age). Animal experiments were approved by the Toronto Centre for Phenogenomics Animal Care Committee.

### Peripheral nerve injury model

Spared nerve injury was performed as described previously [15, 25]. Briefly, mice were anesthetized by isoflurane inhalation and the left sciatic nerve exposed under aseptic conditions. The distal trifurcation of the sciatic nerve was identified and the tibial and common peroneal branches ligated and cut, leaving the sural branch intact. The wound was sutured closed and the animals allowed to recover and returned to their housing.

### von Frey Testing

Von Frey testing for mechanical allodynia following SNI was performed as described previously [18, 26]. Briefly, von Frey fibers were applied to the lateral aspect of the plantar hindpaw in a separate cohort of adult B10, C57BL/6J, A/J, BALB/c and CD-1 mice (n = 6 per strain, except for CD-1: n = 11). The up-down Dixon was used to estimate 50% withdrawal thresholds. In later experiments, an automated von Frey test (UgoBasile Dynamic Plantar Aesthesiometer) was used. Three separate threshold determinations were made on each hindpaw, and then the measurements were averaged.

### Magnetic resonance imaging

A multi-channel 7.0 T, 40 cm diameter bore magnet (Varian Inc. Palo Alto, CA) and 3 cm inner diameter Millipede RF coils (Varian NMR Systems, Palo Alto, CA) were used to acquire in vivo 2D transverse T1-weighted MR images of the thoracic and lumbar spinal cord in multiple mice simultaneously [27]. The imaging protocol consisted of a spin echo sequence with the following parameters: TR = 500 ms, TE = 10 ms, slice thickness = 3 mm, NEX = 8, field-of-view (FOV) = 3.5 × 3.5 cm and matrix size = 350 × 350, producing an image with 100 μm in-plane resolution and an average signal-to-noise ratio of 135 for the spinal cord. The scan time was 23 minutes per slice with three slices acquired sequentially both before and after administration of the contrast agent. Slice localization was accomplished using a sagittal MR image and effort was made to avoid positioning the slices through the lungs to minimize movement artifacts. Mice were injected with a bolus of Gd-DTPA intraperitoneally (1.5 mmol/kg) and the imaging session began 45 minutes after the injection. Mice were imaged using 1–1.5% isoflurane and kept warm during the scan using warm air, with breathing monitored continuously according to established protocols.

### Evans blue assay

Blood spinal cord permeability was determined by Evans Blue extravasation into the spinal cord in a separate cohort of male B10, C57BL/6J, A/J, BALB/c and CD-1 mice (five SNI and five controls) 24 hours after injury. Evan’s Blue dye (2%, 4 mL/kg, Sigma-Aldrich) was infused through the tail vein of anesthetized mice. After two hours animals were perfused with PBS. The spinal cords were immediately dissected and the dura mater removed. The lumbar and thoracic spinal cord tissue were further dissected, weighed and incubated in formamide (Sigma-Aldrich) at 60Â°C for 72 hrs. The Evan’s Blue concentration was then determined by spectrophotometry at 620 nm in a 96-well plate reader.

### Data analysis

Regions-of-interest (ROIs) were chosen to include the entire spinal cord. For each animal, ROIs were measured for one thoracic and two lumbar segments of the cord. Quantitative measurements of signal intensity were determined by normalizing the spinal cord intensity to the average intensity of two phantoms included in each coil (microcapillary tubes containing 1% Gd-DTPA in agar). When the difference in signal intensity between the two phantoms was greater than 10%, attributed to damage to the phantoms or B_1_ field inhomogeneity, the data set was excluded from the analysis. Approximately 15% of measured data was excluded. The ROI placement and signal assessment was performed manually by two investigators and the results averaged. Interobserver variability was expressed as the percent discrepancy between two measurements.

### Statistical analysis

All statistical tests were performed using R statistical software (http://www.r-project.org). For the longitudinal study, a Kruskal-Wallis test was used to determine if there were differences between the treatment groups over time, followed by *t*-tests to compare the groups at each time point. To fit the data, a linear mixed effects model was used. For the study of strain differences, data from both DCE-MRI and the Evans Blue assay was analyzed using *t*-tests to compare the effect of treatment group for each strain and using a two-way ANOVA with treatment group (SNI or control) and strain (B10, C57BL/6J, A/J, BALB/c or CD-1) as between-subject variables. Data from each group of animals are reported as the mean ± standard error of the mean (SEM).

## Electronic supplementary material

Additional file 1: **Before administration of contrast agent, there is no difference in normalized spine signal intensity.** The normalized spine signal intensity in SNI (red) and control (blue) treatment groups before administration of contrast agent for the longitudinal study in CD-1 mice (left panel) and for the study of strain differences (right panel). The mean data is averaged over the thoracic and lumbar ROIs. The error bars illustrate 90% confidence intervals determined by bootstrap resampling of ROI intensity values. The black lines and grey shaded regions illustrate the mean and standard deviation of the combined data. (ZIP 346 KB)

## Abbreviations

B10: B10.D2-Hc^o^H2^d^H2-T18^c^/oSnJ
BSCB: Blood-spinal cord barrier
CD-1: Crl:CD1(ICR)
CSF: Cerebrospinal fluid
DCE-MRI: Dynamic contrast-enhanced magnetic resonance imaging
EAE: Experimental autoimmune encephalomyelitis
FOV: Field-of-view
PNI: peripheral nerve injury
ROI: Regions-of-interest
SNI: Spared nerve injury.
